# The Infrapatellar Fat Pad as a Source of Perivascular Stem Cells with Increased Chondrogenic Potential for Regenerative Medicine

**DOI:** 10.5966/sctm.2016-0040

**Published:** 2016-08-05

**Authors:** Paul Hindle, Nusrat Khan, Leela Biant, Bruno Péault

**Affiliations:** ^1^ MRC Centre for Regenerative Medicine, The University of Edinburgh, Edinburgh, United Kingdom

**Keywords:** Pericytes, CD34+, Chondrogenesis, Adult stem cells, Adipose

## Abstract

Perivascular stem cells (PSCs) are the natural ancestors of mesenchymal stem cells (MSCs) and are the stem cells responsible for homeostasis and repair in vivo. Prospectively identified and isolated PSCs have demonstrated increased plasticity and osteogenic potential. Cells from the infrapatellar fat pad (IFP) have demonstrated increased chondrogenic potential compared with those from subcutaneous fat. This research assessed the chondrogenic potential of IFP PSCs compared with MSCs from the IFP and bone marrow. Immunohistochemistry demonstrated the location of perivascular markers (CD146, CD34, neural/glial antigen 2 [NG2], platelet‐derived growth factor receptor‐β [PDGFRβ], and α‐smooth muscle actin [α‐SMA]) in relation to endothelial markers (CD31, CD144, von Willebrand factor [vWF]). Pericytes and adventitial cells were isolated from the stromal vascular fraction (3.8% and 21.2%, respectively) using flow cytometry with a viability of 88%. The mean numbers of pericytes and adventitial cells isolated were 4.6 ± 2.2 × 10^4^ and 16.2 ± 3.2 × 10^4^, respectively, equating to 7.9 ± 4.4 × 10^3^ and 20.8 ± 4.3 × 10^3^ cells per gram of harvested tissue. Fluorescence‐activated cell sorting demonstrated that cultured PSCs were CD44+CD90+CD105+; polymerase chain reaction and immunocytochemistry demonstrated that pericytes retained their CD146+ phenotype and expressed the pericyte markers PDGFRβ and NG2. Differentiation was confirmed using histochemical stains and genetic expression. Using a pellet model, the IFP PSCs and the MSCs generated significantly more extracellular matrix than bone marrow MSCs (*p* < .001 and *p* = .011, respectively). The IFP PSCs generated significantly more extracellular matrix than IFP MSCs (*p* = .002). Micromass culture demonstrated that differentiated PSCs were upregulated compared with MSCs for *COL2A1*, *ACAN*, and *SOX9* expression by factors of 4.8 ± 1.3, 4.3 ± 0.9, and 7.0 ± 1.7, respectively. The IFP was a significantly better source of chondrogenic stem cells compared with bone marrow. PSCs generated significantly more extracellular matrix than culture‐derived MSCs. Stem Cells Translational Medicine *2017;6:77–87*

**Significance Statement**

This paper presents data on the use of the infrapatellar fat pad as a source of perivascular stem cells for tissue engineering. It also presents quantitative data on the chondrogenic potential of perivascular stem cells and their superiority over culture‐derived MSCs.

## Introduction

Perivascular stem cells (PSCs) have been identified as natural ancestors of culture‐derived mesenchymal stem cells (MSCs) 1 and are responsible for homeostasis and regeneration in vivo 2. PSCs consist of pericytes, which typically reside around smaller blood vessels such as capillaries, venules, and arterioles, and adventitial cells, which are found around larger arteries and veins 1, 3. It has been demonstrated that adventitial cells can produce pericytes; when either of these cell populations are placed in culture, they lead to cell populations that are indistinct from what are commonly termed MSCs 2.

PSCs have shown osteogenic, chondrogenic, adipogenic, and myogenic potential, but this has only been quantified for osteogenesis 4 5 6. Pericyte‐like progenitors have been shown to be more plastic with greater immaturity and engraftment potential compared with MSCs 1, 2, 7, suggesting they would be a better source of stem cells for use in tissue regeneration and engineering. Chondrogenesis was demonstrated by Farrington‐Rock et al. using bovine retinal pericytes 1, 3, 8. In humans, the demonstration of the chondrogenic potential of pericytes has been limited to Alcian blue staining on pellet cultures 1, 3; there are no data quantifying or comparing the chondrogenic potential of pericytes to that of culture‐derived MSCs.

**Figure 1:**
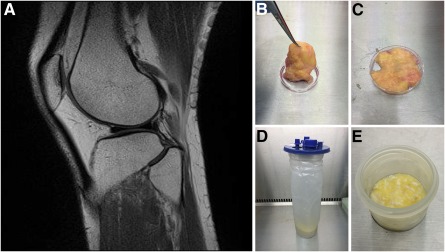
Infrapatellar fat pad location and samples. **(A):** Sagittal magnetic resonance imaging scan of the knee showing the relationship of the infrapatellar fat pad (IFP; white) to the articular cartilage (black). **(B, C):** Excised IFP from a patient undergoing knee arthroplasty **(B)** has the fat removed from the fibrous tissue **(C)**. **(D, E):** The arthroscopically harvested fat pad **(D)** was separated from the irrigation fluid before enzymatic digestion **(E)**.

The in vivo location of chondroprogenitor cells is still debated, with some believing they come from the tissues within the joint and others arguing that they come from subchondral bone. The infrapatellar fat pad (IFP) is an intra‐articular but extrasynovial structure (Fig. 1A) adjacent to the articular cartilage 9. CD146 has been implicated as a marker found on chondroprogenitor cells within articular cartilage 10. Khan et al. identified 3G5‐positive perivascular stem cells on tissue sections of the IFP but found that only a small proportion of cells cultured from the IFP maintained this phenotype 11. The proximity of the infrapatellar fat makes it a candidate for the origin of chondroprogenitor cells. Stem cells derived from the IFP, therefore, could have a greater affinity for cartilage regeneration.

Previous research into PSCs has routinely used subcutaneous fat from the abdomen because this is easily obtained as waste material from patients undergoing elective procedures. The structure and content of adipose tissue varies depending on its location 12, as does the regenerative potential of the harvested MSCs 13, 14. The IFP has demonstrated increased chondrogenic potential compared with subcutaneous abdominal fat from matched patient samples 15. The IFP is also different in that harvesting it from within the joint also includes the synovial membrane.

The authors are not aware of any published data demonstrating that the IFP is a viable source for harvesting PSCs for use in tissue engineering. There are also, to our knowledge, no data quantifying and comparing the chondrogenic potential of PSCs with that of MSCs. Research has typically used IFP samples from patients undergoing knee arthroplasty who are typically in their sixth and seventh decades. Data from these older patients requiring treatment for osteoarthritis may not be applicable to those with focal cartilage defects—the main group suitable for cartilage regeneration procedures and who are typically in their third and fourth decades.

Articular cartilage damage occurs through developmental conditions, trauma, and early degeneration. It typically occurs in younger patients and can cause similar levels of pain and morbidity as end‐stage arthritis 16. This has a significant effect on the patient's ability to work, exercise, and undertake social activities. Articular cartilage defects are incapable of self‐repair and once they reach a critical size, they will degenerate to end‐stage arthritis 17. The cost of treating these problems is estimated to be $40 billion per annum in the U.S. alone. The aims of cartilage repair surgery are to alleviate pain, maximize function of the joint, and prevent progression to end‐stage osteoarthritis. This is of vital importance in young adults because the inevitable degeneration is currently managed with surgical joint replacement, an unattractive option in younger patients because of their high functional demands and the lower longevity of the implant. The ideal treatment for these patients would be regeneration of the hyaline cartilage.

The primary aim of this study was to assess the IFP as a source for prospectively isolating PSCs for tissue engineering, particularly cartilage regeneration. Additional aims were to determine if there were differences in chondrogenic potential between the IFP and bone marrow, and between PSCs and MSCs from the IFP. We also investigated whether samples could be harvested arthroscopically from younger patients and whether these samples differed from those from older patients undergoing arthroplasty, because this has direct implications for orthopedic surgeons harvesting their own samples for cartilage regeneration in the knee.

## Materials and Methods

### Tissue Harvest and Cell Isolation

Collection, storage, and use of tissue samples were approved by the South‐East Scotland Research Ethics Committee (reference no. 10/S1103/45). Infrapatellar fat pad excised as part of total knee replacement (TKR; Fig. 1B) or arthroscopic anterior cruciate ligament reconstruction (ACL; Fig. 1C) were collected and transported to the laboratory in sterile Dulbecco's Modified Eagle Medium (DMEM) supplemented with 10% fetal bovine serum (FBS) and 1% penicillin‐streptomycin (PS).

Tissue samples were mechanically disrupted to ensure they were less than 1 mm^3^ (Fig. 1C, 1E) and were covered with digestion medium (DMEM supplemented with 10% FBS, 1% PS, and 1% collagenase II [Thermo Fisher Scientific Life Sciences, Waltham, MA, http://www.thermofisher.com]). Samples were digested in a shaking water bath at 37°C and 150 rpm for 40 minutes. The digests were mixed with an equal volume of DMEM and then centrifuged at 1,800 rpm for 10 minutes. The supernatant, containing adipocytes and oily fat, was aspirated and discarded. The pellets were resuspended in 25 ml 2% FBS/PBS (5 mM EDTA). The tissue suspensions were successively filtered through a 200‐μm nylon mesh and then 100‐, 70‐, and 40‐μm cell strainers. The strained suspensions were centrifuged at 1,500 rpm for 10 minutes. The supernatant was aspirated and discarded, and the pellets were resuspended for flow cytometry to isolate PSCs. To obtain IFP culture‐derived MSCs, this cell suspension was placed into 10 ml of culture medium in a T75 flask. Bone marrow collected from the distal femur of patients undergoing knee replacement surgery was placed directly into 10 ml of culture medium in a T75 flask to obtain MSCs. The MSC cultures had their medium replaced at 24 hours and all adherent cells were left to proliferate.

### Histology and Immunohistochemistry

Tissue samples were resected so that they were less than 2 cm^3^, to ensure rapid and complete fixation in 4% formalin. Fixed tissue was placed in Tissue‐Tek cassettes (Sakura Finetek, Torrance, CA, http://www.sakura-americas.com) and processed using a Leica ASP processor (Leico Biosystems, Nussloch, Germany, http://www.leicabiosystems.com) using sequential alcohol, xylene, and paraffin wax steps. Tissue was subsequently embedded into molten wax, allowed to solidify on a cold plate, and cut into 7‐μm thick sections. Sections were incubated at 55°C overnight, dewaxed (xylene for 5 minutes, 3 times), and rehydrated (100% ethanol for 2 minutes, 3 times; then 95%, 80%, and 50% ethanol for 2 minutes each; and then washed under running water). Slides were stained as described in the next paragraph, then dehydrated (in 50%, 80%, and 95% ethanol for 30 seconds each, and 100% ethanol for 2 minutes, 3 times), and mounted using xylene.

Sections were incubated in Harris hematoxylin (Sigma‐Aldrich, St. Louis, MO, http://www.sigmaaldrich.com) for 5 minutes and then washed under running water. They were differentiated in 1% hydrochloric acid in ethanol and transferred to a dish of Scott's tap water substitute for 2 minutes until the tissue sections turned blue. Slides were counterstained in eosin for 2 minutes and were washed briefly under running water. Picrosirius red (PSR) solution was made using sirius red F3B (0.1%) in saturated aqueous picric acid. Sections were placed in PSR solution for 2 hours, removed, and washed in running water for 30 seconds.

Tyramide signal amplification was carried out using a Leica BOND‐MAX staining robot (Leica Biosystems). Slides were initially blocked with hydrogen peroxidase (1:10 in BOND wash [BW]) for 10 minutes and then in serum for 10 minutes (type depended on secondary antibody host species). Sections were stained with the primary antibody for 30 minutes (CD31 [catalog no. ab28364], CD34 [catalog no. ab8536], CD146 [catalog no. ab134065], all Abcam, Cambridge, MA, http://www.abcam.com; neural/glial antigen 2 [NG2; catalog no. 554275], BD, Franklin Lakes, NJ, http://www.bd.com; von Willebrand factor [vWF; catalog no. V2700‐01C], US Biological, Salem, MA, https://www.usbio.net) followed by a horseradish peroxidase secondary for 30 minutes (1:50 dilution in serum, goat anti‐rabbit [catalog no. ab7171; Abcam] and goat anti‐mouse [catalog no. ab6823; Abcam]). Tyramide amplification was carried out for 10 minutes (1:50 in own diluent) using fluorescein isothiocyanate (FITC), cyanine (Cy) 3, and Cy5 tyramide kits, depending on the number antibodies required (catalog nos. NEL741B001KT, NEL744B001KT, and NEL745B001KT, respectively; Perkin Elmer, Waltham, MA, http://www.perkinelmer.com) followed by 4′,6‐diamidino‐2‐phenylindole (DAPI) staining for 10 minutes (1:1,000 in BW).

Histochemical staining was imaged using a brightfield setting and color camera with a Zeiss Observer microscope (Zeiss International, Jena, Germany, http://www.zeiss.com). An Olympus BX61 fluorescence microscope (Olympus, Tokyo, Japan, http://www.olympus-global.com) was used to sequentially record the emission from the individual fluorophores used for immunohistochemistry; these were subsequently merged to form a composite image. Controls using isotypes were imaged under identical conditions.

### Flow Cytometry

The cells were blocked in 10% mouse serum in PBS and left at room temperature (RT) for 20 minutes—100 μl for analysis and 500 μl for cell sorting. Antibodies were added to the cell suspension in the dark at a dilution of 1:100 unless otherwise stated. The following antibodies from BD were used for cell sorting: CD31‐PE (BD340297), CD34‐FITC (BD555821), CD45‐PE‐Cy7 (BD557748), and CD146‐AF647 (BD562230). The additional antibodies used when assessing cultured cells included CD34‐PE (catalog no. R1725; Agilent Technologies; Santa Clara, CA, http://www.agilent.com); and from BD: CD44‐AF700 (BD561289), CD56‐PE‐Cy7 (BD560916), CD90‐FITC (BD555595), CD105‐PE‐Cf594 (BD562380), and CD144‐PerCP‐Cy5.5 (BD561566). The cells were incubated for 20 minutes on ice in the dark. The cells were washed in 2 ml of PBS and centrifuged at 1,000 rpm for 5 minutes. The supernatant was aspirated and discarded, and the pellet resuspended in 2% FBS/PBS, with 300 μl used for analysis and 500 μl for cell sorts.

For each antibody, a compensation tube was set up using a single drop of positive compensation beads (BD) diluted with 70 μl of 2% FBS/PBS and stained in an identical manner to the cells. These were resuspended in a final volume of 270 μl and a drop of negative control beads was added just before fluorescence‐activated cell sorting (FACS) analysis or sorting. Gates were set using negative controls to determine the level of true‐positive staining.

### Cell Culture

FACS sorted cells were collected in 300 μl of endothelial cell growth medium (EGM‐2; Lonza, Basel, Switzerland, http://www.lonza.com) as pericytes (CD31−CD34−CD45−CD146+) and adventitial cells (CD31−CD34+CD45−CD146−). Wells and flasks were precoated with 0.2% (weight per volume) gelatin (EMD Millipore, Billerica, MA, http://www.emdmillipore.com) for 10 minutes at 4°C. Cells were plated at a density of approximately 4 × 10^4^ per cm^2^ in EGM‐2 and cultured at 37°C, 5% CO_2_. The EGM‐2 medium was changed after 7 days and then every 4 days after that until the cells reached 90%–100% confluence. From passage 1 onward, all cells were cultured in DMEM/20% FBS/1% PS. Cells were washed twice in PBS and then incubated in 0.05% trypsin‐EDTA at 37°C, 5% CO_2_ for 3–5 minutes until >90% of the cells were mobilized. Complete medium was added to the plate or flask and the cell suspension transferred to a 15‐ml centrifuge tube. The cells were pelleted by centrifuging at 1,000 rpm for 5 minutes. The supernatant was aspirated and discarded, and the pellet was resuspended for further culture expansion, differentiation, or flow cytometry.

### Mesenchymal Differentiation

Monolayer differentiation was undertaken using 20 wells of a 24‐well plate: 10 wells were used for growth medium (DMEM/10% FBS/1% PS) and 10 for differentiation medium (HyClone AdvanceSTEM osteogenic and adipogenic media; GE Healthcare Biosciences, Pittsburgh, PA, http://www.gelifesciences.com). Each set of 10 wells was split into 5 for ribonucleic acid (RNA) extraction and 5 for histochemical staining. The cells were diluted to a concentration of 2 × 10^4^ per ml and 1 ml was added to each well. Plates were incubated at 37°C, 5% CO_2_, until they were 50%–70% confluent, at which point they were considered to be at day 0 of the differentiation course. Plates were taken at day 0 as controls. The medium was removed from those plates undergoing differentiation and replaced with 1 ml of either growth (DMEM/10% FBS/1% PS) or differentiation medium. The media were changed twice weekly for 21 days.

Three‐dimensional pellet culture was used for chondrogenic differentiation. After cell counting, the cells were resuspended in either chondrogenic medium (HyClone AdvanceSTEM; GE Healthcare Biosciences) or DMEM/10% FBS/1% PS at a concentration of 6 × 10^5^ cells per ml, and 500 μl was pipetted into an individual well of a 96‐well V‐bottom plate. Cells were pelleted by centrifugation at 800 rpm for 5 minutes, followed by incubation at 37°C, 5% CO_2_. Six pellets were used for growth medium controls and six for differentiation medium. Of the six, three were used for RNA extraction and three for histochemical staining. The medium was changed twice weekly by centrifuging the plates at 800 rpm for 5 minutes, and then the medium was aspirated, discarded, and replaced with fresh medium. After medium changes, the pellets were gently agitated to ensure they were not adherent.

The micromasss cultures were based on the methods of Zhu et al. 18. The micromasses were formed by resuspending 5 × 10^5^ cells in 30 μl of chondrogenic medium described by Kafienah et al. 19. The cell suspension was gently pipetted into the middle of a single well of a 24‐well plate. The cells were left overnight at 37°C, 5% CO_2_, before adding an additional 1 ml of chondrogenic medium. The medium was changed every 2–3 days for 21 days.

### Histochemistry

For immunocytochemistry, the PBS was removed and the cells permeabilized with 0.05% Triton‐PBS for 3 minutes at RT; the cells were washed three times with PBS and then blocked with Protein Block (Agilent Technologies) for 1 hour at RT. Cells were washed in PBS and then incubated with the primary antibody overnight at 4°C. The following morning, the wells were washed 3 times for 5 minutes—once with PBS‐Tween and twice with PBS. Cells were incubated with the secondary antibody for 1 hour at RT in the dark. After a further three washes, the coverslips were removed and any excess fluid removed. The coverslips were mounted onto slides using DAPI fluoromount and allowed to air dry for 1 hour in the dark before being fixed in place with an adhesive. Cultured pericytes from five different patients were analyzed using three different anti‐CD146 antibodies (clone OJ79c [catalog no. MCA2141F], BioRad Laboratories, Hercules, CA, http://www.bio-rad.com; clone 541‐10B2 [catalog no. 130‐092‐851], Miltenyi Biotec, San Diego, CA, http://www.miltenyibiotec.com; clone P1H12 [catalog no. 563186], BD Biosciences).

Osteogenic differentiation was determined by using Alizarin red to form a birefringent complex with calcium deposits. Alizarin red solution was made by adding 2 g Alizarin red S (C.I. 58005) with 100 mL of distilled water (dH_2_O). This was thoroughly mixed and the pH altered to 4.1–4.3 with 10% ammonium hydroxide. The wells were stained with 1 ml of Alizarin red solution for approximately 30 minutes at RT to produce red‐orange staining with calcium. Excess stain was removed by washing four times with dH_2_O.

Adipogenic differentiation was assessed using Oil Red O. A stock solution was prepared by mixing 0.7 g of Oil Red O (catalog no. Sigma O‐062; Sigma‐Aldrich) with 200 ml of isopropanol. This was stirred overnight and then passed through a 0.2‐μm filter. The stock solution was stored at 4°C. The working solution was made by adding six parts stock solution to four parts dH_2_O. This was mixed and left at RT for 20 minutes before being passed through a 0.2‐μm filter. The wells were washed twice with dH_2_O and then dehydrated with 60% isopropanol for 5 minutes at RT. The isopropanol was removed and the wells dried without washing. The cells were stained with 1 ml of Oil Red O solution for 10 minutes at RT. Excess stain was removed by washing four times with dH_2_O.

Chondrogenic differentiation was indicated by Alcian blue staining for proteoglycans. Slides or wells were incubated with acetic acid for 3 minutes at RT followed by Alcian blue for 30 minutes at RT. Excess stain was removed using acetic acid and the slides were washed three times with dH_2_O. Picrosirius red was also used to determine collagen deposition.

### RNA Extraction

RNA was extracted using TriZol (Thermo Fisher Scientific Life Sciences) and phase separation followed by an RNeasy Micro Kit (Qiagen, Hilden, Germany, https://www.qiagen.com). Samples were stored in TriZol at −80°C so they could be analyzed under identical conditions. Samples were thawed on ice and 200 μl of chloroform was added per 1 ml of TRIzol; these were shaken by hand for 20 seconds, left at RT for 2–3 min and centrifuged at 12,000 rpm and 4°C for 20 minutes. The top, clear layer containing the RNA (approximately 200 μl) was transferred to an RNase‐free 2‐ml Eppendorf tube and were then processed using the RNeasy Micro Kit. The quantity and quality of RNA were measured using NanoDrop (Thermo Fisher Scientific Life Sciences) with an RNA to protein ratio of 1.8–2.0 equating to good quality RNA.

Superscript III reverse transcriptase was used to convert RNA to complementary deoxyribonucleic acid (cDNA). A total of 500 ng to 5 μg was diluted to a total volume of 12 μl with RNAse‐free water. Then 1 μl of random primers and 1 μl of 10 mM deoxynucleotide mix were added. The mixture was denatured at 65°C for 5 minutes and cooled on ice for at least 1 minute. A cDNA synthesis mix consisting 4 μl of first‐strand buffer (5× concentration; Thermo Fisher Scientific Life Sciences), 1 μl of 0.1M dithiothreitol, and 1 μl of Superscript III reverse transcriptase enzyme (Thermo Fisher Scientific Life Sciences). cDNA was synthesized by incubation at 25°C for 10 minutes, 50°C for 60 minutes, and termination of the reaction by heating to 70°C for 15 minutes. The cDNA was cooled to 4°C and kept refrigerated for short‐term use and at −20°C for longer‐term storage.

### Polymerase Chain Reaction

PCR primers were obtained from Bioline (London, U.K., http://www.bioline.com) as a lyophilized powder and were reconstituted to a stock concentration of 100 μM. A working concentration of 10 μM was made by adding 5 μl of left and 5 μl of right primers to 90 μl of RNase‐free water. PCR was performed using MyTaq DNA polymerase (Bioline). The reaction mixture consisted of 5 μl of MyTaq Reaction Buffer (5× concentration), 2 μl of cDNA, 1 μl of primers (10 μM; final concentration, 0.4 μM), 0.25 μl of MyTaq DNA polymerase, and 16.75 μl of RNase‐free water.

The following primers were used for cell identity: *CD31* (F:GAAGTACGGATCTATGACTCA, R:GTGAGTCACTTGAATGGTGCA); *CD34* (F:CATCACTGGCTATTTCCTGAT, R:AGCCGAATGTGTAAAGGACAG); *CD45* (F:CATGTACTGCTCCTGATAAGA, R:GCCTACACTTGACATGCATAC); *CD146* (F:AAGCAACCTCAGCCATGTCG, R: CTCGACTCCACAGTCTGGGAC); *NG2* (F:GCTTTGACCCTGACTATGTTG, R:TCCAGAGTAGAGCTGCAGCA); platelet‐derived growth factor β (*PDGFRβ*; F:CAGTAAGGAGGACTTCCTGGA, R:CCTGAGAGATCTGTGGTTCCA); and β‐actin (*ACTB*; F:CCTCGCCTTTGCCGATCC, R:GGAATCCTTCTGACCCATGC). The additional primers used for differentiation were as follows: collagen II (*COL2A1*; F:GGAAACTTTGCTGCCCAGATG, R:TCACCAGGTTCACCAGGATTGC); *SOX9* (F:ACATCTCCCCCAACGCCATC, R:TCGCTTCAGGTCAGCCTTGC); aggrecan (*ACAN*; F:TGCGGGTCAACAGTGCCTATC, R:CACGATGCCTTTCACCACGAC), hyaluronan and proteoglycan link protein 1 (*HAPLN1*; F:CAACCAGTGCCTGTGTTTGG, R:TATTGGTCCCTGTGGGGTCT); superficial zone protein (*SZP*; F:CTCCTTTTTACAGCAAGGGCG, R:ATTATCCAGCCCGCTTCCAG); runt‐related transcription factor 2 (*RUNX2*; F:ACTGGGCCCTTTTTCAGA, R:GCGGAAGCATTCTGGAA); alkaline phosphatase live/bone/kidney (*ALPL*; F:TCAGAAGCTCAACACCAACG, R:GTCAGGGACCTGGGCATT); osteocalcin (*BGLAP*; F:GCCTTTGTGTCCAAGC, R:GGACCCCACATCCATAG); and peroxisome proliferator‐activated receptor γ (*PPARG*; F:TGAATGTGAAGCCCATTGAA, R:CTGCAGTAGCTGCACGTGTT).

A 100‐ml aliquot of 2% agarose gel was used to run PCR products with a molecular weight (MW) of less than 1 kb. Gels were prepared by dissolving 2 g of agarose in 100 ml of 1× TAE buffer (Tris base, acetic acid, and EDTA; TAE, 10× concentration, diluted 1:10 in dH_2_O; Thermo Fisher Scientific Life Sciences) by heating in the microwave oven until all the agarose had dissolved. Then 10 μl of Gel Red was added (10,000× concentration [catalog no. 41003; Biotium, Freemont, CA, https://biotium.com]). The gel was loaded into a gel‐casting tray; sample combs were inserted and allowed to solidify at RT. The tray was placed in an electrophoresis tank, covered in 1× TAE, and the combs removed. The cDNA samples were diluted to 10 μl with RNase‐free water, mixed with loading dye (2 μl, 6× concentration), and pipetted into a sample well. A low MW DNA ladder was run to allow the identification of band sizes. The electrophoresis was run at 110V for 1 hour.

### Quantitative Real‐Time PCR

Quantitative real‐time PCR was carried out using a Lightcycler 480 (Roche Life Sciences, Indianapolis, IN, https://lifescience.roche.com). cDNA was inserted into a 384‐well plate in triplicate (2 μl per well). Primer‐specific mixtures were created for all samples containing the following: 5 μl of SYBR green master mix (2× concentration; Roche Life Sciences), 2 μl of primers (right and left, 5 μM diluted to a final concentration of 1 μM), and 1 μl of RNase‐free water. The target gene primers used for quantitative real‐time PCR (qPCR) were as follows: collagen I (*COL1A1*; F:GGAACACCTCGCTCTCCA, R:GGGATTCCCTGGACCTAAAG); *COL2A1* (F:CAGAGGGCAATAGCAGGTTC, R:AGTCTTGCCCCACTTACCG); *SOX9* (F:GTACCCGCACTTGCACAAC, R:TCTCGCTCTCGTTCAGAAGTC); and *ACAN* (F:CCTCCCCTTCACGTGTAAAA, R:GCTCCGCTTCTGTAGTCTGC). Three reference genes were tested to determine which was the most stable: glyceraldehyde 3‐phosphate dehydrogenase (*GAPDH*; F:AGCCACATCGCTCAGACAC, R:GCCCAATACGACCAAATCC); hypoxanthine‐guanine phosphoribosyl transferase (*HPRT*1; F:GTAGCCCTCTGTGTGCTCAA, R:TCACTATTTCTATTCATGCTTTGATG), and *ACTB* (F:ATTGGCAATGAGCGGTTC, R:CGTGGATGCCACAGGACT). Then 8 μl of the primer mix was added to each of the wells. The plate was sealed using a sealing foil and stored at 4°C before analysis (less than 2 hours). The qPCR run protocol consisted of an initial preincubation of 95°C for 5 minutes followed by 45 amplification cycles (95°C for 10 seconds; 60°C for 10 seconds; 72°C for 5 seconds with a single detection). Melt curve analysis was run by heating from 65°C to 97°C with 5 acquisitions per degree centigrade.

### Statistical Analyses

All statistical analyses were performed using Statistical Package for the Social Sciences (version 21; IBM, Armonk, NY, http://www.ibm.com). A *p* value less than .05 assumed significance.

## Results

### Histology and Immunohistochemistry

**Figure 2:**
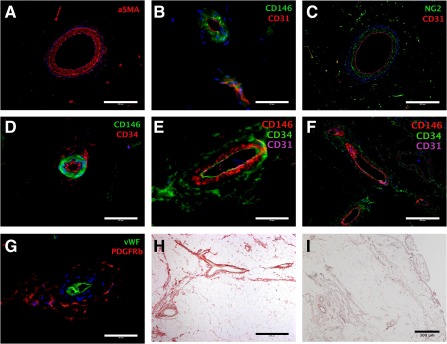
Immunohistochemistry and histology of the infrapatellar fat pad. Sections demonstrated perivascular staining of α smooth muscle actin **(A)**, CD146 **(B, D–F)**, NG2 **(C)**, CD34 **(D–F)**, and PDGFRβ **(G)**. **(B, C, E–G):** The relationship to endothelial markers CD31 and vWF is shown. **(D–F):** The locality of CD146 and CD34 is demonstrated. **(H, I):** Sections were stained with Picrosirius red **(H)** and hematoxylin and eosin **(I)** to demonstrate the perivascular structures **(H, I)** as well as the synovial membrane **(I)**. DAPI was used for nuclear staining and is shown in blue in all images. Scale bar = 200 μm **(A, C, F)**; 50 μm **(B, D, E, G)**; 400 μm **(H)**; and 300 μm **(I)**. Abbreviations: aSMA, α‐smooth muscle actin; NG2, neural/glial antigen 2; PDGFRβ, platelet‐derived growth factor receptor‐β; vWF, von Willebrand factor.

On tissue sections obtained from a patient undergoing a total knee replacement, adipocytes appeared pale as the lipid they contained was dissolved during tissue processing. The remaining cell membranes had a mesh‐like appearance. Small capillaries ran between these cell membranes, with larger vessels, with walls containing smooth muscle, interspersed throughout the adipocytes. The synovial membrane was situated at the right‐hand side of the tissue (Fig. 2I). The synovium was villous and contained numerous synoviocytes at its surface with a rich supply of blood vessels. The sections stained with Picrosirius red showed collagens concentrated around the larger blood vessels (Fig. 2H).

Tissue sections from the same sample were used to document the in vivo location of perivascular cell markers in relation to each other and endothelial cell markers in the infrapatellar fat pad (Fig. 2A–2G). CD31 and vWF were used as endothelial cell markers. CD146 staining was adjacent and abluminal to the CD31 staining. CD34 was also found adjacent and abluminal to the CD146 staining and was also coexpressed with CD31 on the endothelium. The perivascular location of NG2 and PDGFRβ staining was also confirmed. The anti‐PDGFRβ antibody stained the adventitia similar to anti‐CD34 but not the endothelium. Controls imaged under identical conditions for each of the antibodies did not demonstrate any positive staining

### Cell Analysis and Sorting; Variations With Patient Demographics and Conditions

**Figure 3:**
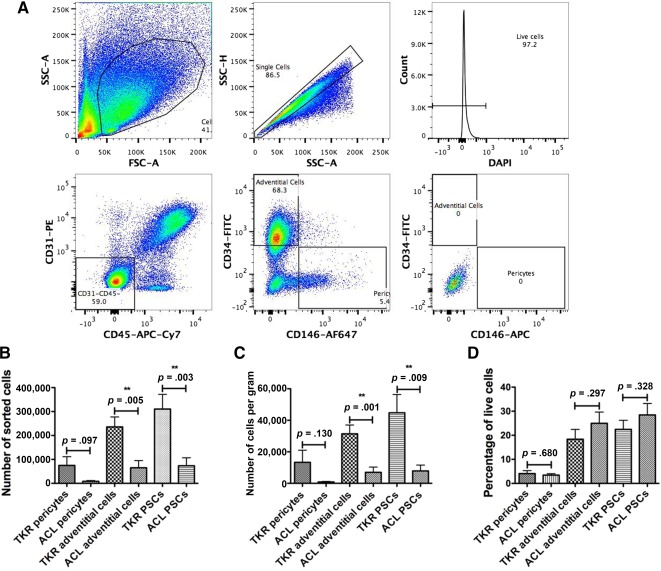
Flow cytometry isolation of perivascular stem cells. **(A):** Fluorescence‐activated cell sorting images demonstrate the gating strategy used to isolate perivascular stem cells. Events likely to represent single cells were selected on their size and relative dimensions, followed by dead cell exclusion using 4′,6‐diamidino‐2‐phenylindole (DAPI; top row). Endothelial cells and hematopoietic stem cells were excluded based on expression of CD31 and CD45, respectively (bottom left). Gates used to select pericytes (CD146+CD34−) and adventitial cells (CD146−CD34+; bottom middle) were based on isotype controls (bottom right). **(B):** Mean number of pericytes, adventitial cells, and perivascular stem cells sorted using fluorescence‐activated cell sorting from patients undergoing ACL reconstruction or TKR were compared. **(C):** The number of cells sorted per gram of tissue were compared between patients undergoing ACL reconstruction or TKR. **(D):** The percentage of the live cells from the stromal vascular fraction that were pericytes or adventitial cells were compared. Abbreviations: ACL, anterior cruciate ligament; APC, allophycocyanin; Cy7, cyanine 7; FITC, fluorescein isothiocyanate; PE, phycoerythrin; SSC‐A, side scatter‐area; SSC‐H, side scatter‐height; TKR, total knee replacement.

**Figure 4:**
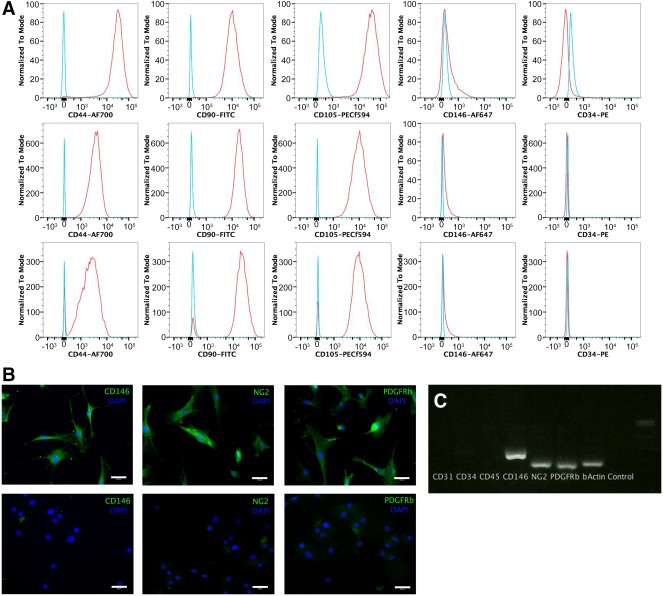
Characterization of cultured perivascular stem cells. **(A):** Cultured pericytes, adventitial cells, and mixed perivascular stem cells were analyzed for expression of mesenchymal and PSCs markers using flow cytometry; left to right: CD44, CD90, CD105, CD146, CD34. **(B):** Cultured pericytes were analyzed using immunocytochemistry for antibodies associated with pericytes (top row). Controls omitted the primary antibodies (bottom row). **(C):** Cultured pericytes were genotyped using reverse transcription polymerase chain reaction. Scale bar = 400 μm. Abbreviations: FITC, fluorescein isothiocyanate; NG2, neural/glial antigen 2; PDGFRβ, platelet‐derived growth factor receptor‐β; PE, phycoerythrin; PSC, perivascular stem cell.

An initial FACS analysis of the stromal vascular fractions of the IFP from patients undergoing total knee arthroplasty and arthroscopic fat pad debridement demonstrated the presence of both pericytes and adventitial cells. The analysis from a patient undergoing a knee replacement is shown in Figure 3. Initial gating used the forward and side‐scatter profiles to select individual cells of the appropriate size and granularity. DAPI was used to exclude dead cells. The tissue processing and analysis were conducted with minimal delay (less than 4 hours from harvest), and the cell viability was 97%. Anti‐CD31 and CD45 antibodies were used to label endothelial and hematopoietic cells, respectively; only cells negative to both antibodies were gated for further analysis. Pericytes and adventitial cells were then selected on the expression of CD146 and CD34 (pericytes CD146+CD34− and adventitial cells CD146−CD34+). Negative controls were used to set the level of true‐positive staining for each of the fluorophores; an unstained sample is shown to demonstrate where these gates were set (Fig. 4A, bottom right plot).

In addition to the 2 analyses, perivascular stem cells were prospectively isolated from 23 patients using fluorescence‐activated cell sorting. Thirteen were from patients undergoing TKRs and 10 were arthroscopically collected during ACL reconstruction. The patients’ mean age (±SEM) was 52.4 ± 4.4 years (TKR, 68.1 ± 3.1 years; ACL, 31.9 ± 3.3 years); mean body mass index was 31.9 ± 1.6 kg/m^2^ (TKR, 36.0 ± 2.0 kg/m^2^; ACL, 27.4 ± 1.8 kg/m^2^). There were 12 male patients and 11 female patients (male‐to‐female ratios: TKR, 4:9, ACL 8:2). The ACL patients were significantly younger and had a lower BMI than patients in the TKR group (independent *t* test, *p* < .001 and *p* = .005, respectively). There was also a significant difference in sex (chi‐square test, *p* = .035).

The data on the amount of harvested tissue and the number and percentages of PSCs harvested are displayed in Table 1 and Figure 3. Although there was a numerical difference in the mass of tissue harvested by the two methods, this did not reach statistical significance (*p* = .066). The numbers of pericytes and adventitial cells were greater in the samples collected from patients undergoing TKR, although this only reached statistical significance for the adventitial cells and when the cells were combined. To ensure that these differences were not related to the mass of tissue collected, the number of cells sorted per gram was also analyzed and demonstrated the same pattern as the results for the total number of cells.

**Table 1:** Patient demographics and fluorescence‐activated cell sorting data

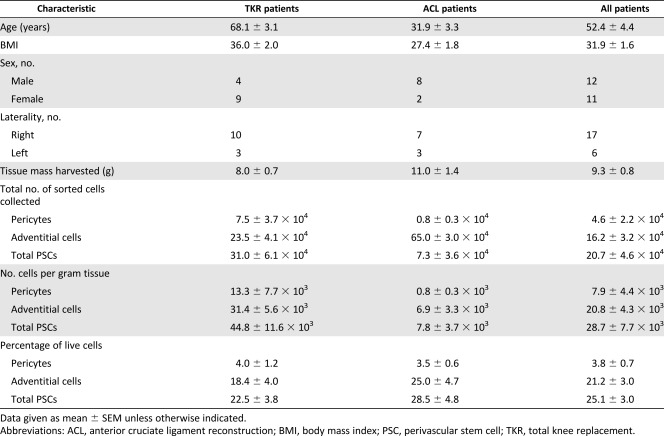

### Phenotype of Cultured Perivascular Stem Cells

Cells were purified using FACS and cultured for between four and six passages in monolayer culture before analysis. Single cells were gated and histograms of each antibody were compared with the relevant isotype control (Fig. 4). Endothelial, hematopoietic, and myogenic cell markers were all absent (CD144, CD45, and CD56 respectively; data not shown). Each of the cell types was positive for the MSC markers CD44, CD90, and CD105. CD34 is not expressed in cultured cells and was absent in all of the tested groups. CD146 is normally expressed by pericytes and late‐passage adventitial cells (unpublished data) but was absent in all of the groups. The compensation beads used to set up the experiment had been strongly positive, demonstrating that the anti‐CD146 antibody was working and the FACS was set up correctly. Subsequent analysis of cultured pericytes (passages 4–6) from 5 separate patients did not demonstrate any CD146 expression compared with isotype controls (data not shown).

Cultured pericytes demonstrated expression of CD146, NG2, and PDGFRβ with immunocytochemistry, compared with controls (Fig. 4). Extracted RNA was reverse transcribed and demonstrated expression of CD146, NG2, and PDGFRβ on gel electrophoresis after PCR (Fig. 4). The RNA‐free control as well as the CD31, CD34, and CD45 reactions were all negative. This demonstrated that cultured pericytes were CD146+ by immunocytochemistry and PCR, but not by FACS.

### Osteogenic and Adipogenic Differentiation

**Figure 5:**
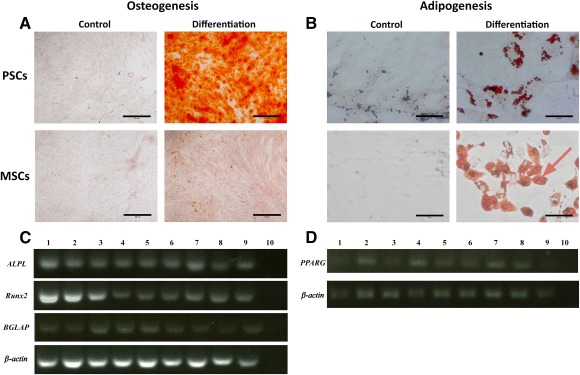
Osteogenic and adipogenic differentiation. **(A):** Osteogenic differentiation of PSC demonstrated increased Alizarin Red staining compared with MSC controls. **(B):** Adipogenic differentiation of PSCs and MSCs demonstrated increased intracellular vesicles compared with growth media controls, which stained positive for Oil Red O. **(C):** Differentiated cells expressed RUNX2, ALPL, and BGLAP; *ACTB* was used as the reference gene. **(D):** Differentiated cells expressed PPARG; *ACTB* was used as the reference gene. Scale bar = 50 μm. Channels used in polymerase chain reaction images, left to right: 1, anterior cruciate ligament (ACL) pericytes; 2, ACL adventitial cells; 3, ACL mixed PSCs; 4, total knee replacement (TKR) pericytes; 5, TKR adventitial cells; 6, TKR mixed PSCs; 7, ACL MSCs; 8, TKR MSCs; 9, bone‐marrow‐derived MSCs; 10, complementary deoxyribonucleic acid negative control. Abbreviations: MSC, mesenchymal stem cell; PSC, perivascular stem cell.

Cultured pericytes, adventitial cells, and mixed PSCs, from both ACL and TKR samples, all exhibited osteogenic, adipogenic, and chondrogenic potentials. Osteogenic differentiation was confirmed with functional staining with Alizarin red for calcium deposition and with genetic expression of runx‐2, ALPL, and BGLAP confirmed on PCR (Fig. 5). Adipogenic potential was confirmed by intracellular uptake of Oil Red O in lipid vacuoles and genetic expression of pPARγ (Fig. 5).

### Chondrogenic Potential of PSCs From IFP Compared With That of MSCs

**Figure 6:**
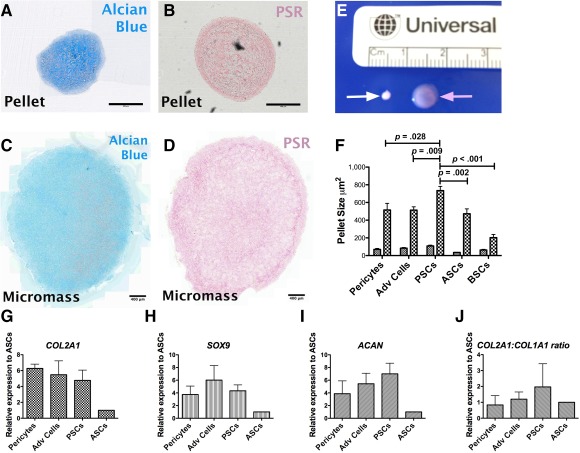
Chondrogenic differentiation of PSCs. Chondrogenesis was confirmed using pellet **(A, B)** and micromass **(C, D)** models indicated by Alcian Blue staining **(A, C)** for sulfated proteoglycans and Picrosirius Red **(B, D)** for collagens I and II. **(E):** Macroscopic difference in appearance is seen between the pellets (white arrow) and micromasses (pink arrow). **(F):** PSCs created significantly larger pellets compared with mesenchymal stem cells from the fat pad and bone marrow. Relative genetic expression of chondrogenic markers indicated that infrapatellar fat pad (IFP) PSCs had greater expression of COL2A1 **(G)**, SOX9 **(H)**, and ACAN **(I)**, as well as an increased ratio of COL2A1 to COL1A1 **(J)** compared with IFP MSCs. Scale bar = 400 μm **(A–D)**. Abbreviations: Adv, adventitial; ASC, adipose‐derived stem cell; BM‐MSC, bone marrow‐derived mesenchymal stem cell; PSC, perivascular stem cell; PSR, Picrosirius red stain.

Chondrogenic differentiation was confirmed with histochemical stains of proteoglycans with Alcian blue and collagens with Picrosirius red (Fig. 6). A cartilage pellet model demonstrated that both the IFP PSCs and MSCs generated significantly more extracellular matrix than bone marrow‐derived MSCs (*p* < .001 and *p* = .011, respectively; Fig. 6). Pericytes, adventitial cells, and combined PSCs were compared with adipose‐derived MSCs (also known as adipose‐derived stem cells [ASCs]) and bone marrow‐derived MSCs (BM‐MSCs). Each cell type was cultured using growth medium (Fig. 6F, left columns) and chondrogenic medium (Fig. 6F, right‐hand columns). The PSCs generated significantly more extracellular matrix than the IFP MSCs (ASCs; *p* = .002).

A micromass culture demonstrated clear Alcian blue and Picrosirius red staining on sections, indicating the production of proteoglycans and collagens (Fig. 6). qPCR showed that differentiated PSCs were upregulated compared with MSCs for *COL2A1*, *ACAN*, and *SOX9* expression by factors of 4.8 ± 1.3, 4.3 ± 0.9, and 7.0 ± 1.7, respectively (Fig. 6).

## Discussion

This research has accurately identified pericytes and adventitial cells within the human infrapatellar fat pad. Previous identification of pericytes within the IFP used a monoclonal anti‐3G5 antibody, which stained the tunica adventitia of a small arteriole 11. Pericytes are classically described as being found around smaller vessels 1; the histochemical staining presented by Khan et al. was similar to the peri‐arteriolar reactivity we observed with an antibody to α‐SMA (Fig. 2A). The immunohistochemistry in Figure 2B clearly demonstrated CD146 staining around the endothelium of a microvessel, in keeping with pericytes. Figure 2E and 2F shows that the pericyte staining was separate from the tunica adventitia where the adventitial cells are located. Our data confirmed the perivascular location of pericytes and adventitial cells within the IFP and that they were distinct cell populations.

CD146 was normally expressed by IFP pericytes in situ (Fig. 2) and following cell dissociation (Fig. 3), but by only extremely few cultured IFP pericytes (Fig. 4). We do not have a definitive interpretation for this observation, which we have made sporadically, whereas CD146 expression is generally sustained in vitro. For additional information, the reader is referred to the paper by Crisan et al. 1. A possible explanation is that CD146, being a cell adhesion molecule, exhibits variable expression depending on cell density or confluence in the culture flask. It is known that state of confluence influences the expression of many cell surface markers in cultures of human bone marrow stromal cells 20.

There is no consensus on the optimal tissue for harvesting stem cells for cartilage repair and regeneration procedures. The most commonly used in registered clinical trials are bone marrow‐derived MSCs. Bone marrow has demonstrated superiority over subcutaneous fat in two studies 21, 22 but no difference in another 23, and synovium has demonstrated superiority over bone marrow 24. Neither of these has been compared with adipose tissue from the infrapatellar fat pad, which has demonstrated superiority over subcutaneous fat 15. Lopa et al. used culture‐derived MSCs with no cell purification or isolation 15. Our data agreed that the IFP was a superior source of stem cells (PSCs or MSCs) compared with bone marrow (Fig. 6F). Furthermore, when we compared PSCs with MSCs from the IFP using relative genetic expression, we found that there was an advantage to isolating PSCs using flow cytometry compared with deriving MSCs from culture of the stromal vascular fraction (Fig. 6G–6J). The increase in relative genetic expression of chondrogenic cell markers was consistent with other studies that have found significant differences when comparing cell sources for chondrogenic differentiation 15.

Articles that have advocated synovium as a source of regenerative cells have not included histology or characterization to indicate which cells were being used 25. The histology and immunohistochemistry images in this study demonstrated how narrow the synovium is and how hard it would be to physically harvest only synovial tissue and not the underlying tissue. The immunohistochemistry demonstrated that the synovium has a rich vascular supply populated with PSCs. Our data suggest that synovium‐derived MSCs originate from these perivascular cells. The synovium is thought to play an active role in immunomodulation within the knee, particularly in osteoarthritis 26. The utility of cells from the synovium is in keeping with Caplan's theory that MSCs work by growth factor and cytokine secretion rather than engraftment and differentiation 27, 28.

We have isolated pericytes and adventitial cells from IFPs as distinct populations for culture, analysis, and differentiation. The cells were also combined immediately after isolation and cultured together to investigate whether there was a synergistic effect, because the two populations are found in close proximity in vivo. No clear distinction was demonstrated during our analysis, but during the cartilage pellet formation, the coculture produced significantly larger pellets than the pericytes or adventitial cells cultured individually. Although these are distinctly different cells, this synergistic effect would prove an interesting subject for further investigation.

Approximately six to eight culture passages were required to get a sufficient number of cells to complete the PSC identity work and the differentiation experiments. This means that the PSCs were from higher‐passage cultures than the MSCs, which were always used at passage 2. This difference would theoretically disadvantage the PSCs in this comparison, suggesting a greater advantage of PSCS over MSCs if used at a comparable passage number. Approximately 1 × 10^6^ cells per cm^2^ are used for autologous chondrocyte implantation 29 and commercial companies typically use passage 3 chondrocytes 30. Proliferation of autologous PSCs from the IFP to clinically relevant numbers would likely require the same number of passages.

Our data also demonstrated that it is possible to obtain viable cells from tissue that has been harvested arthroscopically. This means that orthopedic surgeons can harvest adipose tissue samples without the need for liposuction and could either electively plan to collect IFP using minimally invasive arthroscopy, or harvest tissue opportunistically while performing arthroscopy for another reason.

There are more than 20 clinical trials using MSCs for articular cartilage damage registered with the ClinicalTrials.gov website 31. Our data suggest that PSCs are, in fact, superior to MSCs and that current clinical trials are using suboptimal, uncharacterized cells, which might be a reason for the heterogeneous results reported in the literature with animal and human studies 32. Further work is required to define the optimal cells and their preparation for chondrogenesis if translation to patients is to be given the best chance of being effective.

## Author Contributions

P.H.: conception and design, provision of study material or patients, collection and/or assembly of data, data analysis and interpretation, manuscript writing, final approval of manuscript; N.K.: collection and/or assembly of data; L.B.: conception and design, provision of study material or patients, manuscript writing; B.P.: conception and design, data analysis and interpretation, manuscript writing, final approval of manuscript.

## Disclosure of Potential Conflicts of Interest

L.B. has institutional research support from Sanofi, Vericel, Medacta, Smith and Nephew; owns stock in Ostesys; and is an independent provider of medicolegal report service to injured clients. The other authors indicated no potential conflicts of interest.
